# Supplementary material for: Recovery of Rare Earth Elements from Wastewater Towards a Circular Economy
Source: Molecules. 2019 Mar 13;24(6):1005. doi: 10.3390/molecules24061005 (PMC6471397; doi:10.3390/molecules24061005)
Supplement: Supplementary file 1 [file molecules-24-01005-s001.pdf]

Supplementary data:

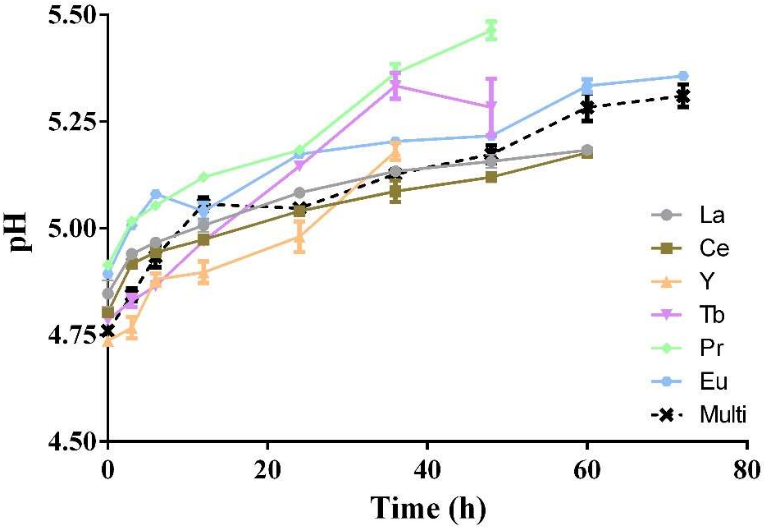

Figure S1: pH variation for the single and multi-solute assays with zeolite and REE, with the respective standard deviation (n=3, X=2).

Table S1: Variation of the pH for the single and multi-solute assays with bacterial biomass, with the respective standard deviation (n=2, X=2).

Time	Single- component						Multi solute
	La	Ce	Eu	Pr	Tb	Y	
**0 h**	4.9 ± 0.12	4.9 ± 0.13	5.0 ± 0.01	4.9 ± 0.06	4.9 ± 0.15	4.9 ± 0.13	5.0 ± 0.00
**48 h**	4.9 ± 0.10	4.9 ± 0.00	5.0 ± 0.00	4.9 ± 0.02	4.9 ± 0.04	4.9 ± 0.03	5.0 ± 0.01

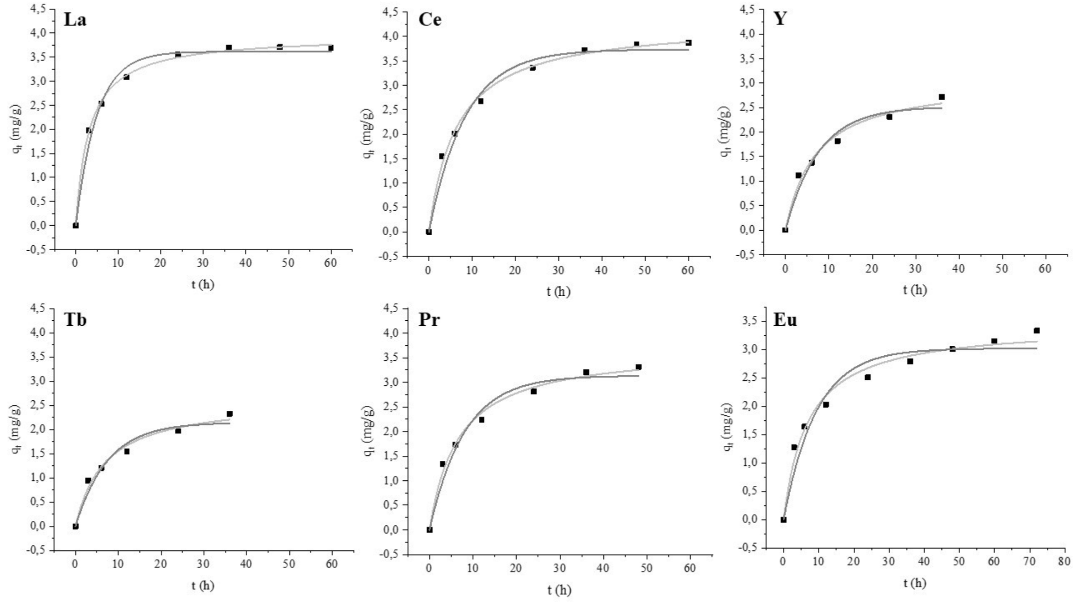

Figure S2: Representation of adsorption capacity at each time, q_t, versus time for each REE (■) in the single solute assay with zeolite for the PFO (——) and PSO (······) modelling (n=3, X=2).

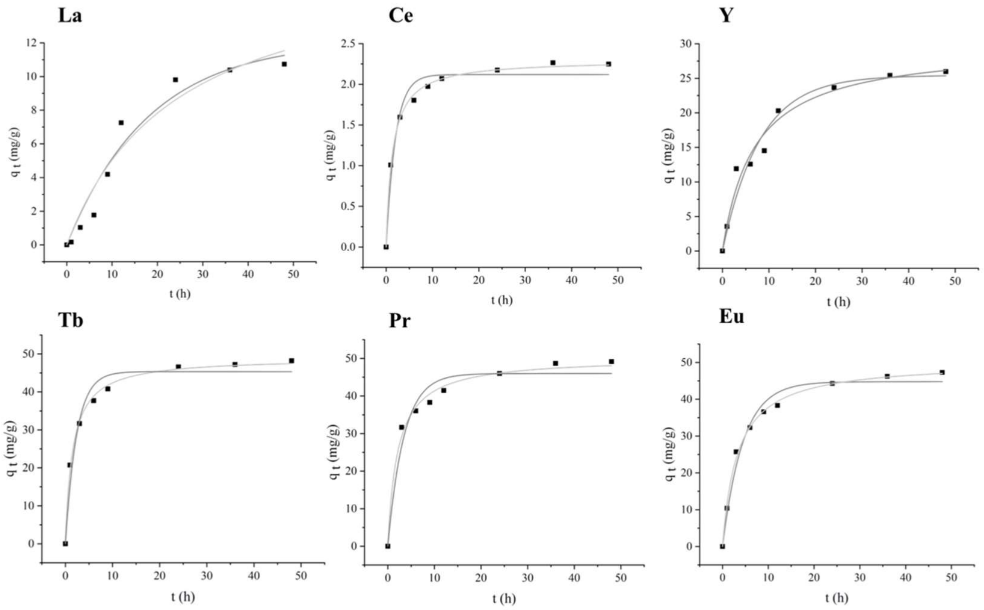

Figure S3: Representation of adsorption capacity at each time, q_t, versus time for each REE (■) in the single component assay with suspended biomass representing PFO (—) and PSO (—) modeling.

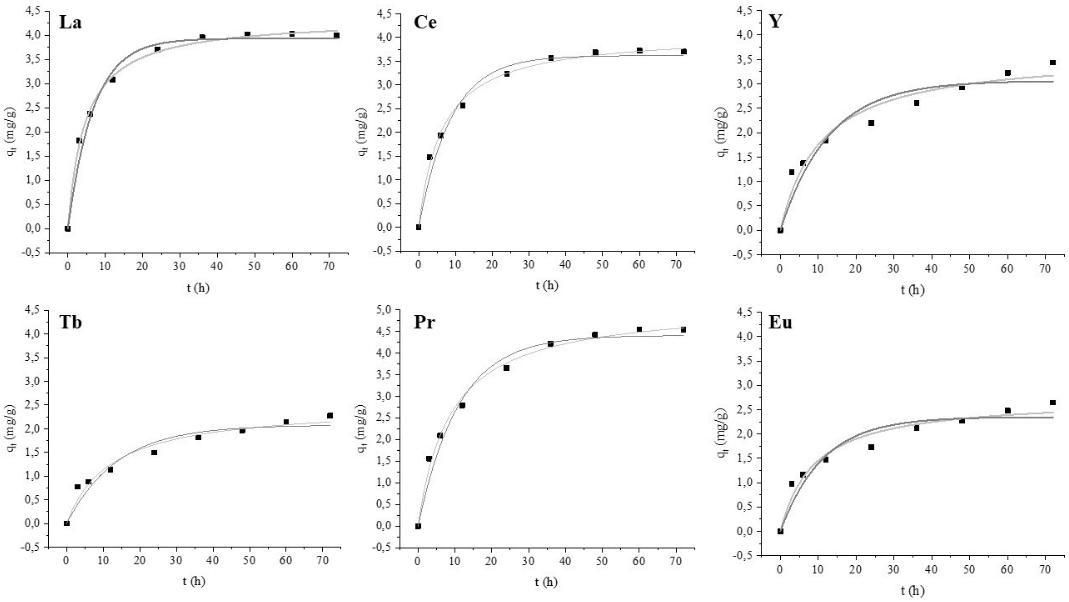

Figure S4: Representation of adsorption capacity at each time, q_t, versus time for each REE (■) in the multi solute assay with zeolite for the PFO (—) and PSO (—) modelling (n=3, X=2).

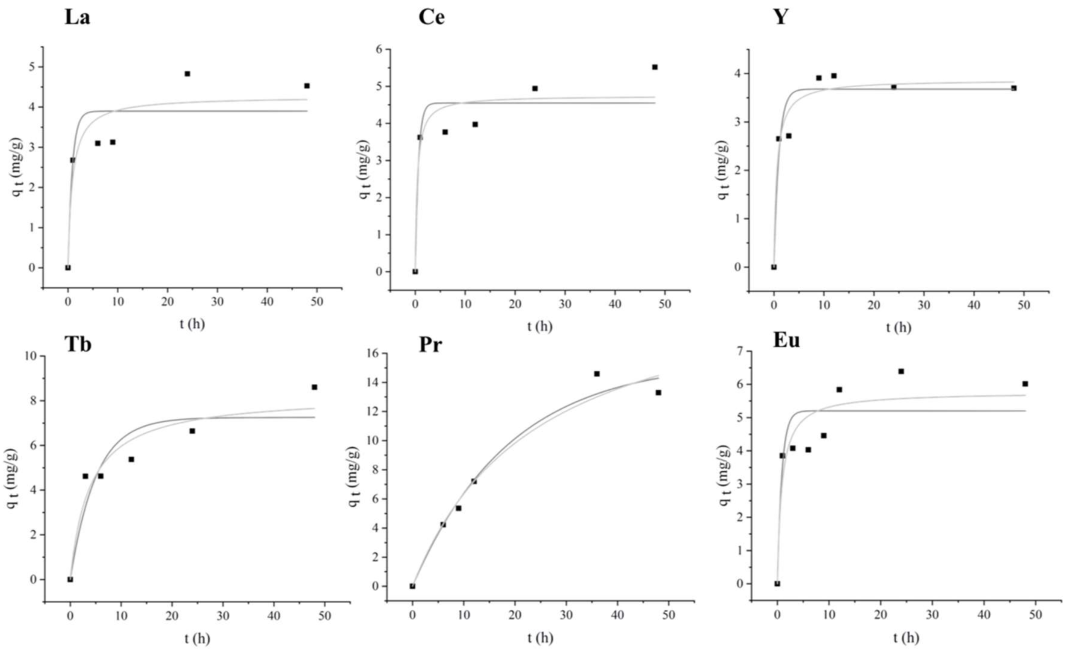

Figure S5: Representation of adsorption capacity at each time, qt, versus time for each REE (■) in the multi solute component assay with suspended biomass representing PFO (—) and PSO (—) modeling.

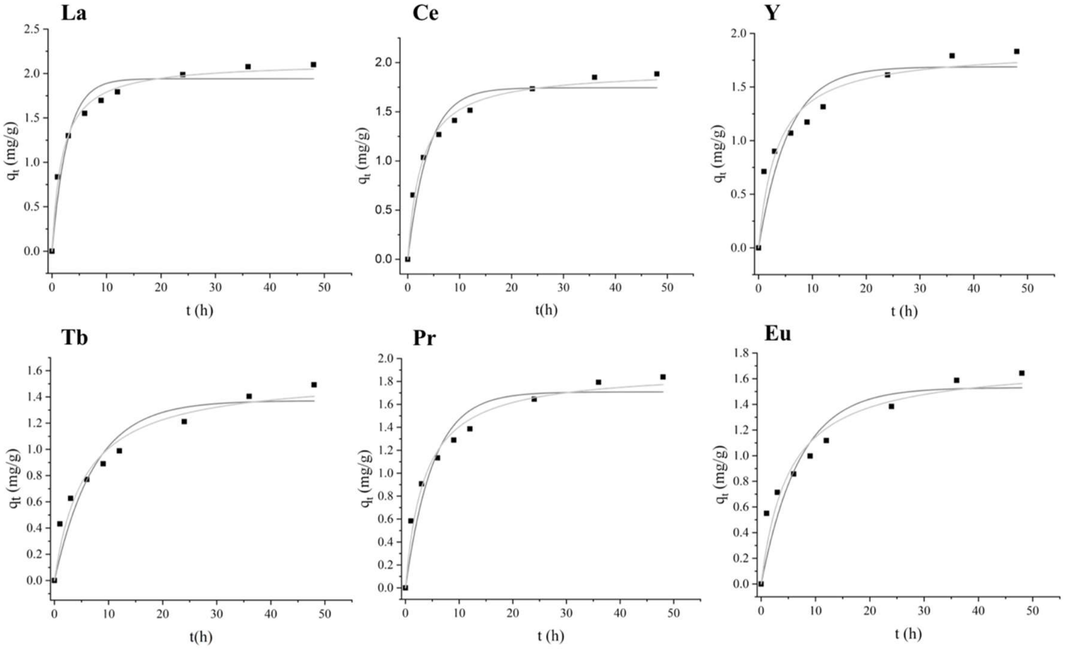

Figure S6: Representation of adsorption capacity at each time, qt, versus time for each REE ( ) in the multi solute component assay with supported biomass on zeolite representing PFO (—) and PSO (—) modeling.

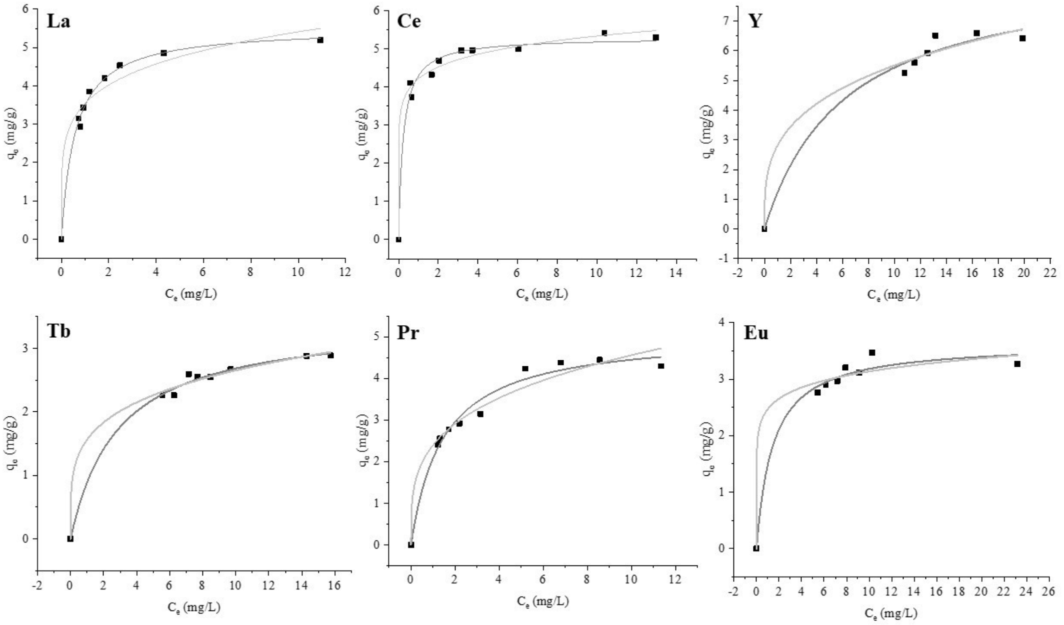

Figure S7: Adsorption isotherms graphs corresponding to Langmuir (▬) and Freundlich (▬) for the tested single solute REE assays with zeolite (n=3, X=2).

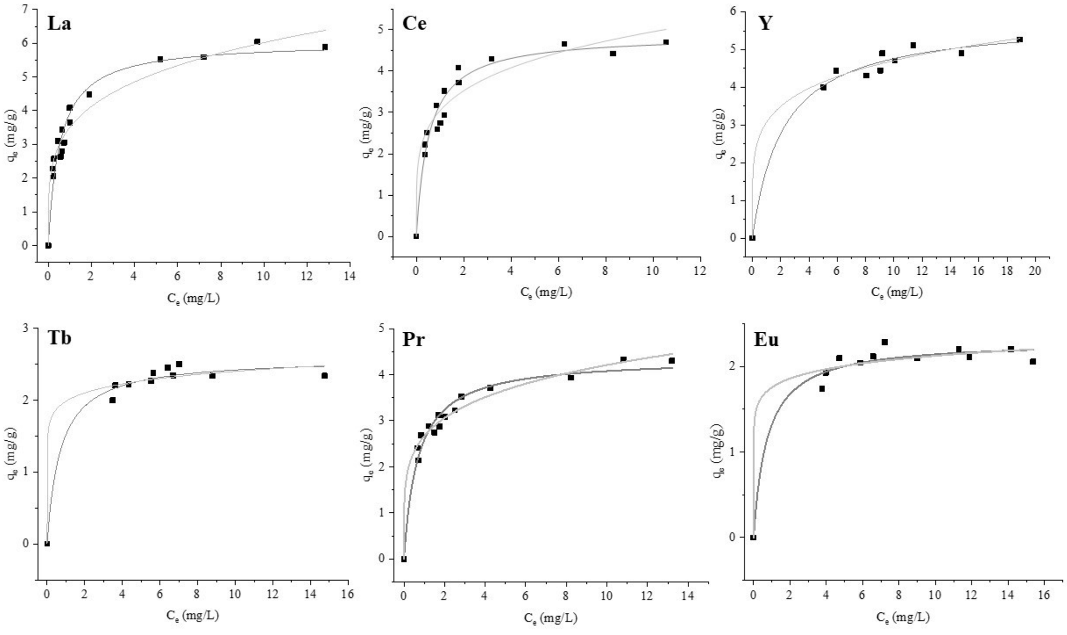

Figure S8: Adsorption isotherms graphs corresponding to Langmuir (▬) and Freundlich (▬) for the tested multi solute REE assays with zeolite (n=3, X=2).

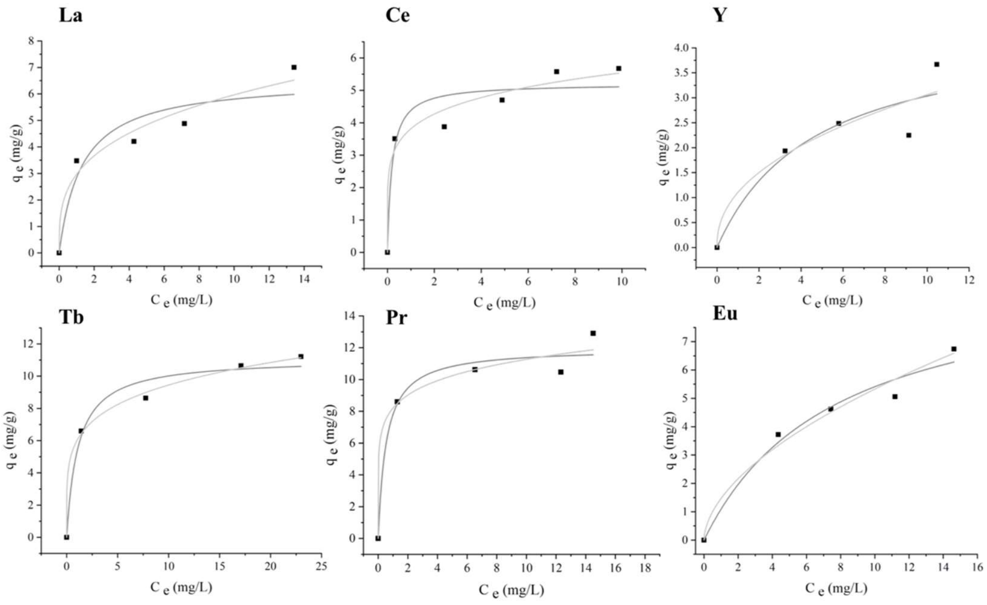

Figure S9: Adsorption isotherm graphs corresponding to Langmuir (▬) and Freundlich (▬) for the tested multiple-component REE with suspended biomass (n=2, X=2).

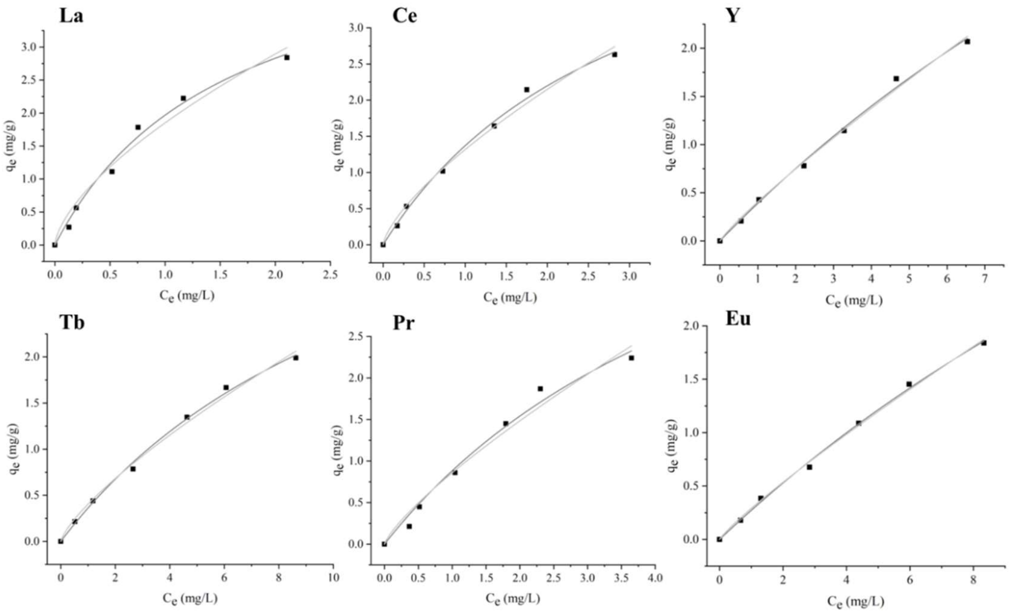

Figure S10: Adsorption isotherm graphs corresponding to Langmuir (▬) and Freundlich (▬) for the tested multiple-component REE with supported biomass on zeolite (n=2, X=2).

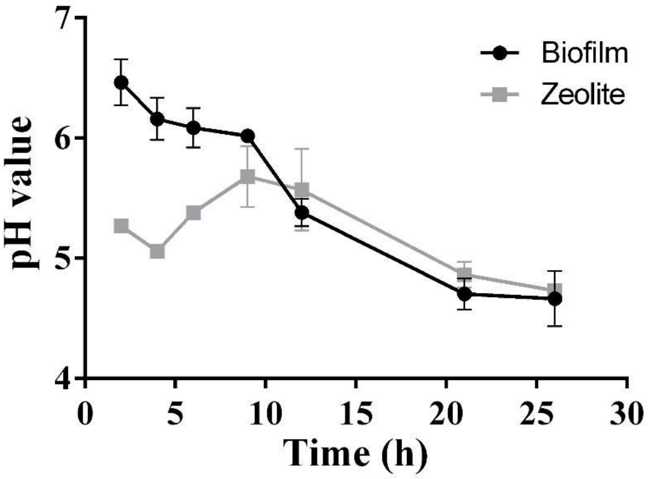

Figure S11: pH variation for the multi solute assays with zeolite with or without biofilm for the column assays, with the respective standard deviation (n=3, X=2).
